# Fulminant Influenza B Myocarditis in an Adolescent Requiring Venoarterial Extracorporeal Membrane Oxygenation (VA-ECMO): Complete Recovery at a Pediatric Cardiac Referral Center in Mexico

**DOI:** 10.7759/cureus.103920

**Published:** 2026-02-19

**Authors:** Chantale Gilles-Herrera, Cindy A Hernández-Cárdenas, Alejandra Gutierrez-Lugo, Gabriela I Pereira-Lopez, Claudia González-García, Antonio Benita-Bordes, Oscar Garcia-Sánchez, Antonio Juanico-Enriquez, Juan Calderon-Colmenero

**Affiliations:** 1 Cardiovascular Pediatric Intensive Care, Instituto Nacional de Cardiología Ignacio Chávez, Ciudad de México, MEX; 2 Pediatric Cardiology, Instituto Nacional de Cardiología Ignacio Chávez, Ciudad de México, MEX; 3 Cardiothoracic Surgery, Instituto Nacional de Cardiología Ignacio Chávez, Ciudad de México, MEX

**Keywords:** extracorporeal membrane oxygenation support, fulminant myocarditis, influenza virus type b, pediatric cardiac icu, viral-induced myocarditis

## Abstract

Influenza is a common viral infection associated with seasonal outbreaks; however, fulminant myocarditis secondary to influenza Type B represents a rare and life-threatening complication in the pediatric population, with poorly understood pathophysiology. Only a limited number of cases have been reported worldwide, and to our knowledge, this is the first published case in Mexico. We report the case of a previously healthy 13-year-old girl who presented with a five-day history of nonspecific symptoms and rapidly progressed to cardiogenic shock. Prompt recognition and a multidisciplinary approach were essential, involving the pediatric cardiology, cardiac surgery, intensive care, and cardiac imaging teams. Advanced mechanical circulatory support was initiated, including an intra-aortic balloon pump and extracorporeal membrane oxygenation. Medical management included antiviral therapy, corticosteroids, and intravenous immunoglobulin. Following 45 days of intensive management, the patient demonstrated complete recovery of cardiac function and was discharged with close outpatient follow-up. This case highlights the importance of early recognition of fulminant myocarditis in pediatric patients with influenza infection and demonstrates that timely multidisciplinary management and advanced mechanical circulatory support can result in favorable outcomes, even in severe presentations.

## Introduction

Fulminant myocarditis (FMC) is a severe and rapidly progressing form of myocarditis that can lead to cardiogenic shock [1,2]. It accounts for 10-30% of myocarditis cases and is implicated in 10-20% of sudden and unexplained deaths in children [3]. 

Pathophysiologically, FMC is driven by an intense inflammatory response within the myocardium, resulting in extensive myocyte necrosis, interstitial edema, and severe ventricular dysfunction [2]. Unlike acute or chronic myocarditis, which may follow a more gradual course with mild symptoms, FMC has a fulminant onset and rapidly leads to profound systolic dysfunction. Due to its critical nature, prompt hemodynamic support, often requiring vasopressors, inotropes, or advanced circulatory assistance, such as extracorporeal membrane oxygenation (ECMO), is essential [2,3].

In Mexico’s latest report on influenza infection incidence from the most recent seasonal outbreak (2023-2024), a total of 6,875 confirmed cases were recorded. Influenza B accounted for 14.6% of these cases, with a fatality rate of 4% among Influenza B infections [4].

While viral infections are the leading cause of FMC, enteroviruses, particularly Coxsackievirus B, are most frequently implicated. Influenza B, though less common, is a clinically significant cause due to its potential for direct myocardial injury and an exaggerated immune response, which can trigger a cytokine storm and circulatory collapse [3]. Given the rarity of severe cardiac involvement in influenza B, early recognition is challenging but critical, as timely pharmacological and mechanical support can be lifesaving. This report presents a pediatric case of influenza B-associated FMC complicated by cardiogenic shock, requiring ECMO support. We describe the clinical course, therapeutic approach, and a review of the literature to highlight the importance of early diagnosis and intervention in this rare but severe manifestation of influenza infection.

## Case presentation

A 13-year-old girl with no significant medical history was admitted to our hospital in cardiogenic shock after a 5-day history of non-specific symptoms, including malaise, facial edema, nausea, vomiting, abdominal pain, and mild exertional dyspnea. Prior evaluation at a pediatric unit revealed ST-segment abnormalities and elevated cardiac enzymes.

On admission, physical examination showed altered mental status, grade II jugular venous distention, weak carotid pulses with low amplitude, generalized hypoventilation attributed to respiratory muscle fatigue secondary to cardiogenic shock, fixed splitting of the second heart sound, and diminished heart sounds. Laboratory evaluation revealed leukocytosis, elevated acute-phase reactants, cardiac enzyme elevation, N-terminal pro-B-type natriuretic peptide (NT-proBNP) of 28,538 pg/mL, and hyperlactatemia (5.6 mmol/L). 

**Figure 1:**
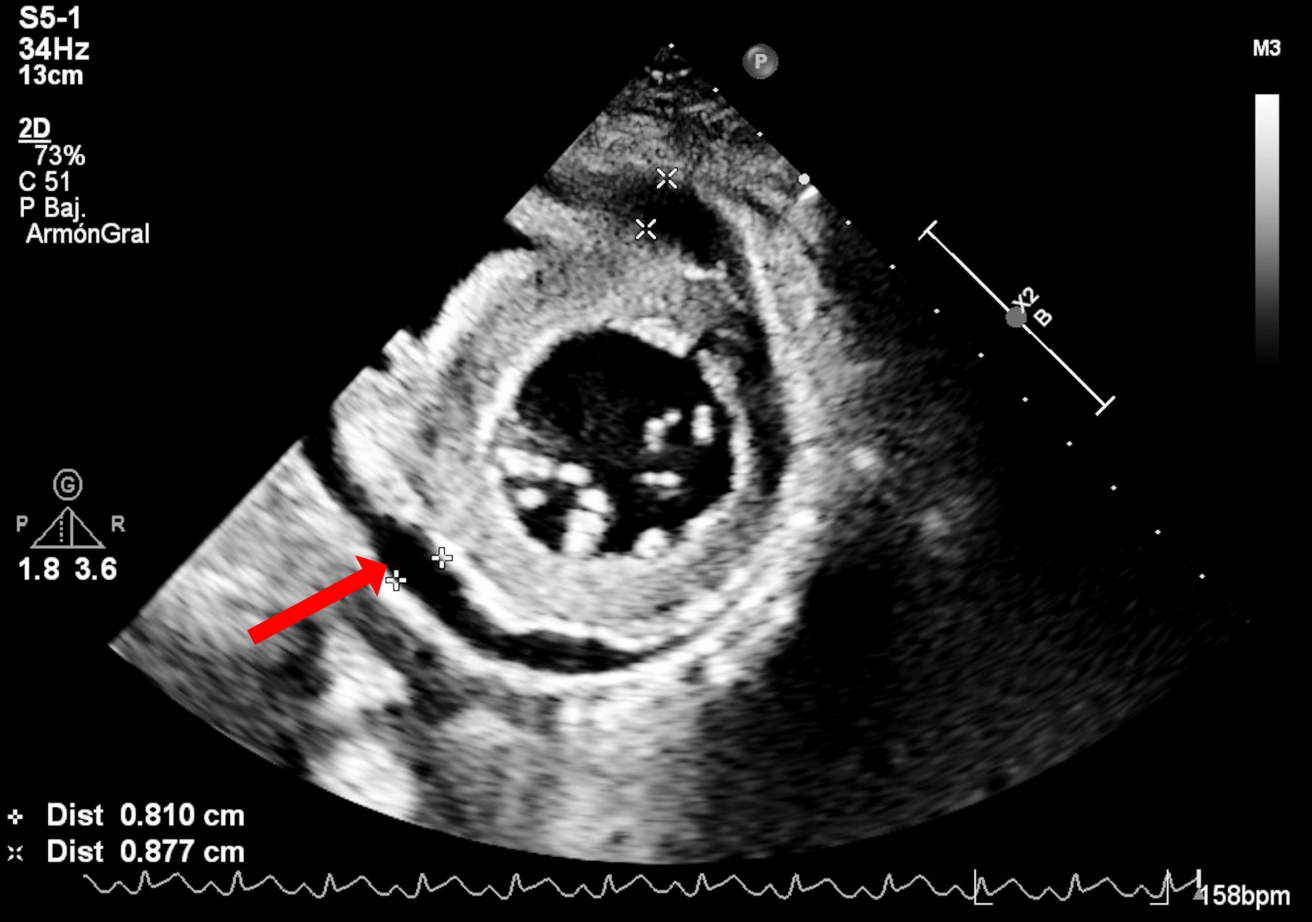
Echocardiogram: parasternal short axis view Pericardial effusion and hyperechogenic myocardium due to inflammation and edema were noted.

**Figure 2:**
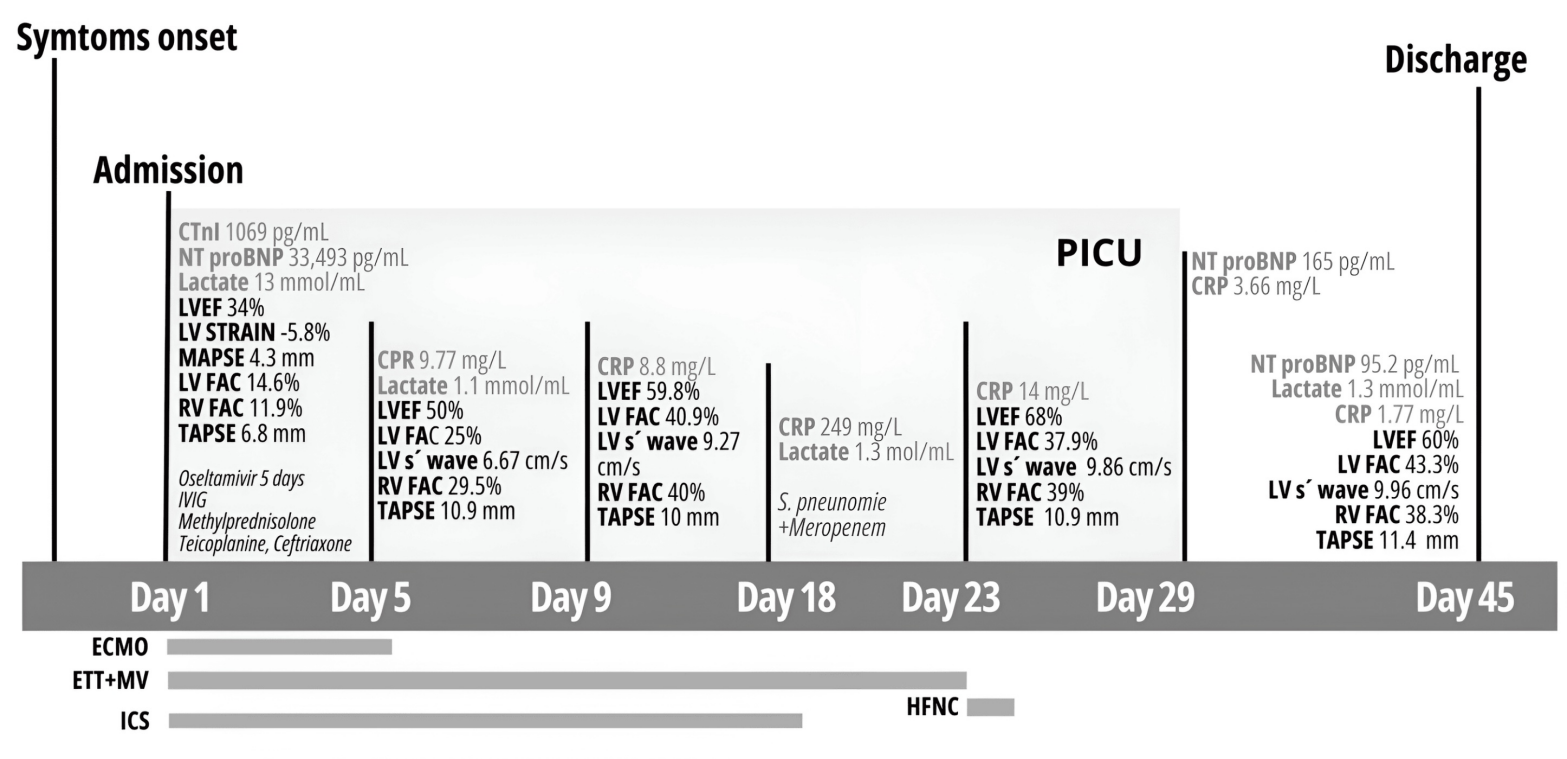
Timeline and echocardiographic evolution during the in-hospital stay CTn-I, cardiac troponin I; NT-pro BNP, 1N-terminal pro B-type natriuretic peptide; CRP, C-reactive protein; LVEF, left ventricle ejection fraction; LV, left ventricle; MAPSE, mitral annular plane systolic excursion; FAC, fractional area change; TAPSE, tricuspid annular plane systolic excursion

Electrocardiography demonstrated sinus tachycardia with diffuse anterior ST-segment elevation (V1-V6), consistent with probable acute pericarditis. Chest radiography showed grade II cardiomegaly with left ventricular enlargement. Transthoracic echocardiography confirmed severe biventricular systolic and diastolic dysfunction with mild circumferential pericardial effusion measuring 8.7 mm (Figure 1). A timeline integrating echocardiographic findings and clinical milestones is presented in Figure 2.

The patient was transferred to the Pediatric Cardiac Intensive Care Unit (PCICU), where vasoactive and inotropic support was initiated, including levosimendan, epinephrine, norepinephrine, and vasopressin. An intra-aortic balloon pump (IABP) was placed, but she progressed to refractory cardiogenic shock (Society for Cardiovascular Angiography & Interventions (SCAI) stage C; inotropic score 57), requiring venoarterial extracorporeal membrane oxygenation (VA-ECMO). Peripheral cannulation was initially performed.

Due to inadequate flow delivery with peripheral cannulation and persistent hypoperfusion manifested by progressive renal and hepatic dysfunction and worsening hyperlactatemia (peak lactate 13 mmol/L), the patient was transitioned to central VA-ECMO (right atrial drainage cannulas 26 Fr and 28 Fr, and 20 Fr aortic return cannula). Organ dysfunction was attributed to inadequate systemic perfusion rather than ECMO-related complications, as rapid improvement in end-organ markers followed restoration of adequate flow. Initial ECMO parameters were a flow of 3.27 L/min at 3,400 rpm (cardiac index 1.9 L/m²/min).

**Figure 3:**
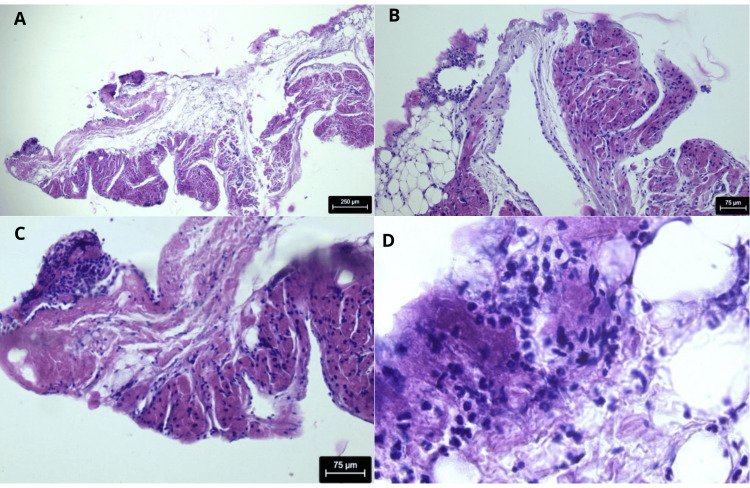
Histopathologic evidence of viral myopericarditis in right atrial appendage biopsy 3A. Hematoxylin and eosin (H&E), 4×. Low-power view showing the relationship between atrial myocardium, subepicardial connective tissue, and visceral pericardium, with multifocal inflammation compatible with myopericarditis. 3B. H&E 10x. of right atrial appendage tissue showing interstitial mononuclear inflammatory infiltrate and edema separating myocardial fibers, consistent with acute myocarditis. 3C. H&E 10x. Epicardial and pericardial tissue demonstrating dense inflammatory infiltrate and connective tissue thickening, consistent with acute pericarditis. 3D. H&E 40x. Revealing predominantly mononuclear inflammatory infiltrate, composed mainly of lymphocytes and macrophages, without granulomas or eosinophilic infiltration, supporting the diagnosis of viral myocarditis.

During ECMO cannulation, a biopsy of the right atrial appendage was obtained. Histopathological examination revealed atrial myocardial tissue with interstitial mononuclear inflammatory infiltrates, edema, and focal myocyte injury, consistent with acute myocarditis. In addition, inflammatory involvement of the visceral pericardium was identified, consistent with acute pericarditis. No myocardial necrosis was observed. Given the atrial sampling site, these findings reflect atrial and epicardial inflammation and do not allow accurate assessment of the extent of ventricular myocardial involvement (Figure 3). In parallel, multiplex real-time polymerase chain reaction testing (FilmArray® Respiratory Panel, BioFire, Salt Lake City, Utah, US) was performed at admission on an endotracheal aspirate obtained during intubation for ECMO support. Influenza B was identified 48 hours later, at which time antiviral therapy with oseltamivir, already initiated empirically, was continued, confirming the diagnosis of fulminant influenza B myocarditis. Immunomodulatory therapy included five pulses of methylprednisolone and intravenous immunoglobulin.

Progressive hemodynamic improvement followed, with gradual tapering of inotropic therapy and echocardiographic evidence of ventricular function recovery (Figure 2). The patient was successfully decannulated from central VA-ECMO on day 5, and delayed sternal closure was performed 24 hours later as part of a routine institutional post-ECMO strategy, not due to hemodynamic instability. Inotropic support was fully discontinued 13 days after ECMO removal.

On hospital day 13, *Klebsiella pneumoniae* was isolated from a tracheal aspirate culture. Given clinical deterioration, fever, increased ventilatory requirements, and elevated inflammatory markers, the finding was interpreted as true pneumonia rather than colonization, and meropenem therapy was initiated with a favorable clinical response. The patient was extubated on day 23 of hospitalization.

**Figure 4:**
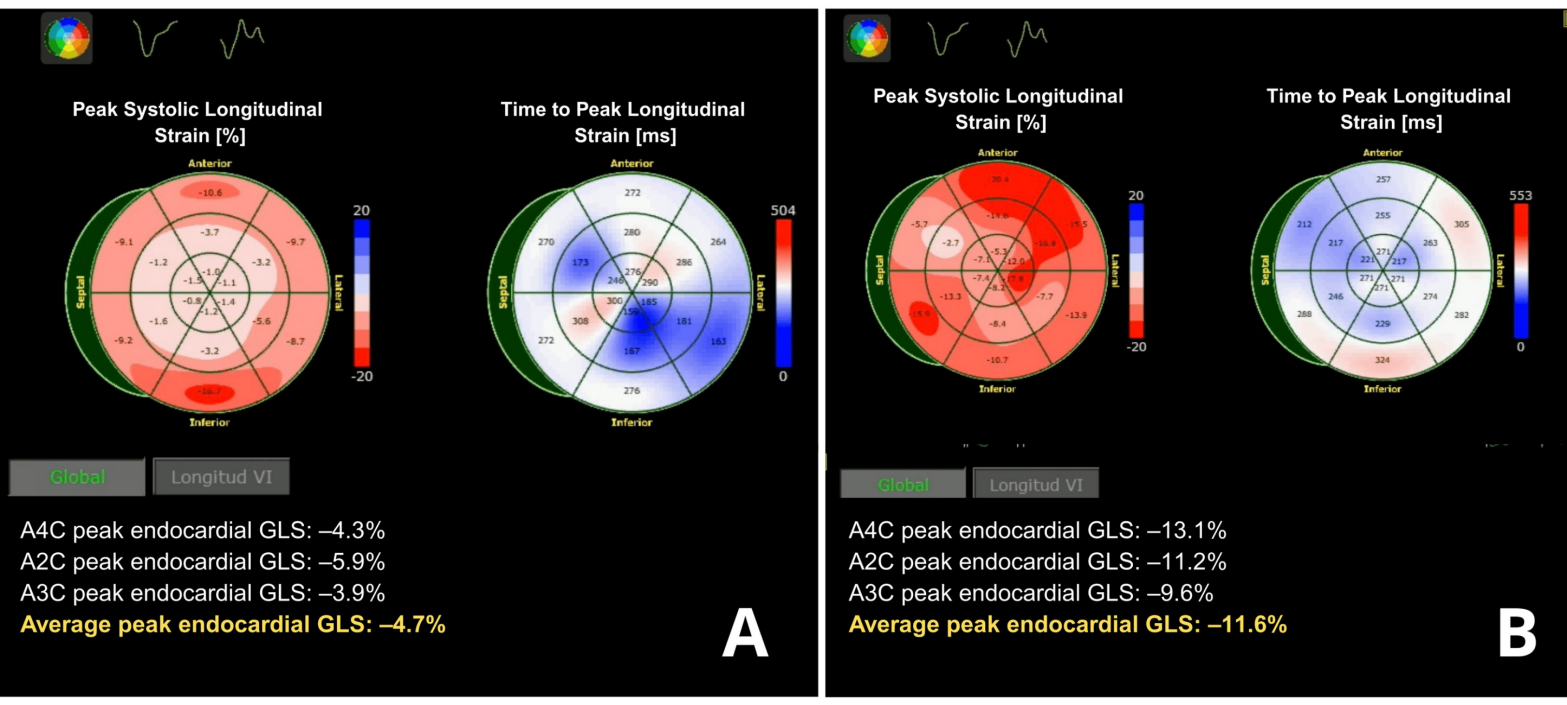
Evolution of global longitudinal strain demonstrating severe initial myocardial dysfunction and subsequent recovery (A) Baseline speckle-tracking echocardiography obtained on admission demonstrating severely reduced peak endocardial global longitudinal strain (GLS), with an average GLS of –4.7%, consistent with severe acute myocardial dysfunction. (B) Follow-up speckle-tracking echocardiography demonstrating marked improvement in myocardial deformation parameters, with recovery of peak endocardial GLS to an average of –11.3%, reflecting substantial functional myocardial recovery following mechanical circulatory support and clinical stabilization.

At discharge on hospital day 45, the left ventricular ejection fraction had normalized; however, global longitudinal strain remained impaired at −11.6% as compared to −4.6% at admission (Figure 4), reflecting ongoing myocardial recovery. She was discharged on carvedilol, furosemide, and digoxin. This therapeutic combination was selected to address persistent functional myocardial impairment by optimizing preload, modulating neurohormonal activation, and supporting contractility during convalescence. Close outpatient follow-up was scheduled.

## Discussion

FMC represents the most severe end of the myocarditis spectrum and differs from non-fulminant forms in its pathophysiology, clinical presentation, and prognosis [1,2]. Histopathologically, FMC is characterized by diffuse inflammatory infiltrates, marked myocardial edema, and acute myocyte injury, leading to abrupt hemodynamic deterioration. Clinically, it presents with rapid progression to severe heart failure or cardiogenic shock, often within days of symptom onset. In contrast, acute or chronic myocarditis typically follows a more indolent course, whereas ACS-like myocarditis in pediatric patients is usually dominated by chest pain, focal ST-segment changes, and relative hemodynamic stability at presentation [5-7]. These distinctions are clinically relevant, as FMC frequently necessitates early escalation to mechanical circulatory support, whereas ACS-like presentations may initially be managed conservatively [1-3,5,6].

Viruses account for approximately 50-70% of myocarditis cases in children [8]. Influenza B-associated myocarditis remains uncommon, and fulminant presentations are exceptional. Epidemiologic surveillance data and multicenter influenza networks highlight the overall low incidence of severe cardiac complications during influenza seasons [4,8]. As summarized in Table 1, only a limited number of pediatric cases with severe cardiac involvement due to influenza B have been reported worldwide [9-17], with a predominance of female patients, frequent gastrointestinal symptoms at presentation, delayed diagnosis, and high mortality rates. To our knowledge, this represents the first reported successful pediatric case of influenza B-associated fulminant myocarditis requiring VA-ECMO with complete myocardial recovery in Latin America to date. Previously reported cases from the region have been limited and included fatal outcomes confirmed by postmortem.

**Table 1:** Case reports of severe cardiac damage associated with influenza B infection in pediatric patients until August 2024 Note: This table summarizes previously reported pediatric cases of influenza B-associated myocarditis, including diagnostic delay (from symptom onset to diagnosis), presenting symptoms, cardiac biomarker levels, ventricular function, treatment strategies, and survival outcomes. These data provide the clinical context for comparison with the present case. Abbreviations: NR, not reported; cTn-I, cardiac troponin I; NT-proBNP, N-terminal pro-B-type natriuretic peptide; EKG, electrocardiogram; LVEF, left ventricular ejection fraction; FAC, fractional area change; PCS, pharmacological cardiac support; ECMO, extracorporeal membrane oxygenation; IVIG, intravenous immunoglobulin; ATG, antithymocyte globulin; CRRT, continuous renal replacement therapy.

Case	Age Sex	Delay diagnosis (days)	Symptoms / Clinical presentation	CTn-I (ng/ml)	NT-pro BNP (pg/ml)	Chest X-ray / EKG	LVEF / FAC (%)	Treatment	Survival
1997, Craver et al. [9], USA	6 F	1	Chest pain, diarrhea, vomiting, lethargy, no fever	NR	NR	Cardiomegaly/ ST-segment elevation	NR	Pharmacological cardiac support (PCS)	No
2004, Tabbut et al. [10], USA	4.5 F	2	Abdominal pain, emesis, lethargy. Fulminant myocarditis and myositis → cardiogenic shock	NR	NR	Mild cardiomegaly/ Normal	NR/ 10%	PCS, ECMO (7 d), IV gamma globulin, plasmapheresis, hemodialysis	Yes
2009, Muneuchi et al. [11], Japan	15 M	2	Sudden chest pain, fever. Mimicking acute coronary syndrome	cTn-T 1.53 mg/ml	NR	NR/ ST-segment elevations	63%/ NR	IV isosorbide dinitrate	Yes
2010, Frank et al. [12], Germany	5 F	1	Abdominal pain, no fever, previous febrile upper respiratory tract infection. Fulminant myocarditis → cardiogenic shock	NR	NR	Normal/ unspecific low-grade alterations with generalized paroxysms, sharp-wave gradients	NR / Severe reduction	PCS (unsuccessful resuscitation attempts)	No
2017, McCormick et al. [13], USA	5 F	NR	Periorbital and facial edema, myalgia, and recent febrile upper respiratory tract infection. Fulminant myocarditis	1180 pg/mL	NR	Normal/ sinus tachycardia, right axis deviation, and nonspecific ST-T wave changes	44%	PCS, Oseltamivir IVIG Methylprednisolone, ECMO (9 d)	Yes
2018, Piccinni et al. [14], USA	13 F	3	Upper respiratory symptoms, chest pain. Fulminant myocarditis → cardiogenic shock	cTn-T 9 Ng/ml	NR	Normal/ ST-segment changes	Severely decreased biventricular function	ECMO (14 d), Oseltamivir, Methylprednisolone, IVIG, ATG	Yes
2021, Arguello [15], Costa Rica	17 F	2	Vomiting, chest pain, dyspnea.	387.8 pg/ml	NR	ST-segment elevation	NR/NR	Unsuccessful resuscitation attempts	No
2023, Puzelli et al. [16], Italy	6 F	5	Vomit, asthenia, abdominal pain, no fever	1416.6 pg/ml	NR	NR/ Low QRS voltage	20%/NR	PCS (unsuccessful resuscitation attempts)	No
2024, Tian et al. [17], China	7 M	3	Fever, projectile vomiting, lethargy, convulsive crisis. Fulminant myocarditis → cardiogenic Shock	169.6 pg/ml	5094 pg/ml	NR/ ST-segment elevation,	38.5% after ECMO	PCS, ECMO (5 d), CRRT, Methylprednisolone, Oseltamivir, Interferon	No
Gilles et al., this study, Mexico	13 F	5	Periorbital edema, malaise, cough, vomiting, abdominal pain. Fulminant myocarditis → cardiogenic Shock	592 pg/ml	28538 pg/ml	Grade II tachycardia/ Sinus tachycardia and extensive anterior ST-segment elevation	34% / 11.3%	PCS, IBP, ECMO (5 d), Oseltamivir, IVIG, Methylprednisolone	Yes

A consistent feature across reported cases is diagnostic delay, with a median time of approximately five days from symptom onset to diagnosis, as described by Puzelli et al. and Tian et al. [16,17]. This delay appears to be driven primarily by nonspecific early manifestations, particularly gastrointestinal symptoms, such as abdominal pain and vomiting, facial or peripheral edema, and unexplained fatigue, often occurring in the absence of prominent respiratory symptoms or high-grade fever. Importantly, most reported patients did not have significant pre-existing comorbidities, consistent with findings from large pediatric FMC cohorts in which major underlying conditions were present in a minority of cases [3]. Currently, no validated early biomarker reliably predicts progression to FMC. While elevated cardiac troponins and NT-proBNP support myocardial involvement, they are frequently obtained only after clinical deterioration has occurred [18]. Cardiac magnetic resonance imaging may facilitate earlier recognition; however, its use is often limited in hemodynamically unstable pediatric patients [19].

Early recognition of clinical patterns suggestive of FMC is therefore essential. In this and previously reported cases, warning signs most commonly included predominant gastrointestinal symptoms, facial or peripheral edema, subtle exertional dyspnea, and rapid clinical deterioration disproportionate to initial findings. In contrast, cardiogenic shock, multiorgan dysfunction, malignant arrhythmias, severe biventricular systolic impairment, and marked hyperlactatemia generally represent later complications resulting from delayed diagnosis and progressive hemodynamic compromise. Awareness of this progression may prompt earlier cardiac evaluation and timely escalation of care before irreversible organ injury develops.

Several predictors of adverse outcome in pediatric myocarditis have been consistently reported, including younger age, female sex, delayed diagnosis, severe ventricular dysfunction, arrhythmias, renal impairment requiring dialysis, and markedly elevated NT-proBNP levels [5-7,20,21]. NT-proBNP reflects myocardial wall stress and neurohormonal activation and has been linked to increased mortality and the need for mechanical circulatory support, particularly at values exceeding 5,000 pg/mL [5,6]. In patients supported with ECMO, evidence of end-organ injury, especially peak serum creatinine, has also emerged as an independent predictor of in-hospital mortality [21]. Beyond their statistical significance, these variables should directly inform bedside decision-making by prompting early consideration of advanced mechanical circulatory support rather than prolonged escalation of pharmacologic therapy alone.

Notably, our patient exhibited multiple poor prognostic factors at presentation, including female sex, initial gastrointestinal symptoms, markedly elevated NT-proBNP levels (>28,000 pg/mL), severely depressed FAC (11.3%), and reduced left ventricular ejection fraction (LVEF). Serial laboratory values and echocardiographic measurements obtained at critical time points during hospitalization are summarized in Table 2 and illustrate several key temporal trends relevant to prognosis: NT-proBNP peaked early during ECMO support and subsequently declined rapidly following ventricular unloading, normalizing prior to discharge; LVEF progressively improved from 34% at admission to 60% before discharge; and serum creatinine, which transiently increased during ECMO, normalized after circulatory recovery. Inflammatory markers similarly demonstrated an early surge followed by resolution.

**Table 2:** Laboratory test and LVEF during in-hospital critical dates Note: Odds ratios refer to the change in odds of poor early outcomes when the predictor variables are present. Abbreviations: CTn-I, cardiac troponin I; NT-pro BNP, N-terminal-pro B-type natriuretic peptide; CPR, C-reactive protein; LVEF, left ventricular ejection fraction

	Admission	ECMO day 1	During ECMO peak level	Off ECMO one week	Previous discharge	Normal range	Early Poor prognosis levels [6]	P value OR (CI 95%) [6]
CTn-I (ng/ml)	592	667	1069	-	-	<0.16	-	-
NT-proBNP (pg/ml)	28538	33493	33493	165	95.2	<125	>5000	0.037, OR 15
CPR (mg/L)	3.17	4.92	39	249	1	0.1-1.0	-	-
Creatinine (mg/dl)	0.7	0.9	1.4	0.6	0.4	0.45-0.81	-	-
LVEF (%)	34%	-	-	50.50%	60%	>60%	<30%	0.006, OR 60

Importantly, several of these high-risk features were present despite the absence of profound systolic depression at presentation, underscoring the limitation of relying on ejection fraction alone to assess disease severity. Although the initial LVEF exceeded 30%, the severity of this case was not explained by systolic impairment alone but rather by the combined burden of diffuse myocardial inflammation, pericardial involvement, and profound circulatory collapse. Acute myocarditis may precipitate shock despite only moderate depression of ejection fraction because inflammatory myocardial edema reduces ventricular compliance, promotes diastolic dysfunction, and induces myocardial stunning, thereby limiting effective cardiac output. Concomitant biventricular involvement and systemic inflammatory vasoplegia may further aggravate hypotension independently of left ventricular systolic performance. In this physiologic context, ejection fraction underestimates true disease severity and should not delay escalation to advanced mechanical support, an approach that likely contributed to the rapid reversal of biomarker abnormalities and ventricular dysfunction observed in this case.

In clinical practice, fulminant myocarditis is initially managed with cautious fluid resuscitation and inotropic support [3,19]; however, rapid progression to refractory cardiogenic shock is common [22]. Nonetheless, the hemodynamic benefit of IABP may be insufficient in the setting of severe biventricular dysfunction, inflammatory myocardial edema, or vasoplegic shock, physiologic hallmarks of FMC.

Percutaneous ventricular assist devices, such as the Impella system (Abiomed, Danvers, Massachusetts, US), have similarly emerged as temporary support options in children and adolescents with cardiogenic shock, including acute myocarditis. These devices may provide short-term left ventricular unloading and serve as a bridge to recovery or to more definitive therapy; however, pediatric experience remains limited to small observational cohorts and case series, and reported outcomes are heterogeneous. In clinical practice, a substantial proportion of pediatric patients supported with Impella ultimately require escalation to more comprehensive modalities, including VA-ECMO or durable ventricular assist devices, particularly when biventricular failure or profound systemic hypoperfusion is present [23,24]. Consequently, in fulminant myocarditis characterized by rapid hemodynamic collapse, multiorgan hypoperfusion, and limited response to pharmacologic therapy or partial unloading strategies, VA-ECMO remains the most appropriate initial mechanical support platform, providing immediate biventricular assistance and full cardiopulmonary stabilization while myocardial recovery is assessed [1-3,25,26].

Survival outcomes for pediatric FMC supported with ECMO have improved over time. Contemporary series and registry analyses report survival rates of 60-75% in experienced centers, reflecting advances in patient selection, cannulation strategies, and multidisciplinary management [6,7,25-27]. In our patient, peripheral cannulation failed to achieve adequate flows, necessitating early transition to central VA-ECMO. Despite multiple poor prognostic factors, including female sex, gastrointestinal symptoms, markedly elevated NT-proBNP levels, renal dysfunction, and severe biventricular involvement, full myocardial recovery was achieved. This favorable outcome likely reflects timely escalation to advanced mechanical support, early correction of inadequate perfusion, and coordinated multidisciplinary care.

However, the ability to implement such strategies is highly dependent on institutional resources and regional systems of care. Centers with established ECMO programs, pediatric cardiac intensive care units, advanced cardiac imaging capabilities, and dedicated multidisciplinary heart failure teams are more likely to achieve early stabilization and timely escalation to mechanical circulatory support. In contrast, limited access to advanced devices, shortages of trained personnel, financial constraints, delayed interhospital transfer, and lack of regional referral networks may substantially prolong time to diagnosis and definitive therapy.

Within this context, this case differs from previously reported series by demonstrating survival without major sequelae in a critically ill adolescent with influenza B-associated fulminant myocarditis in a region where access to advanced mechanical circulatory support is not universally available. Although a single case cannot inform resource allocation decisions, it highlights persistent disparities in access to life-saving therapies and underscores the importance of established referral networks to specialized centers. Our experience therefore reinforces prior reports suggesting that recognition of high-risk clinical patterns and timely escalation of care may substantially influence outcomes in pediatric FMC.

## Conclusions

Clinicians should maintain a high index of suspicion for myocarditis in pediatric patients presenting with nonspecific but concerning features, particularly prominent gastrointestinal symptoms, facial or peripheral edema, unexplained fatigue, and rapid clinical deterioration, even in the absence of marked respiratory complaints or fever.

This case illustrates that complete myocardial recovery is possible in influenza-associated fulminant myocarditis when early recognition, prompt referral, and advanced mechanical circulatory support are achieved, even in regions with limited baseline access to such therapies. Strengthening pediatric cardiac critical care networks and referral pathways may therefore be critical to improving outcomes in similar settings.

## References

[1] Venkataraman S, Bhardwaj A, Belford PM, Morris BN, Zhao DX, Vallabhajosyula S (2022). Veno-arterial extracorporeal membrane oxygenation in patients with fulminant myocarditis: a review of contemporary literature. Medicina (Kaunas).

[2] Ammirati E, Frigerio M, Adler ED (2020). Management of acute myocarditis and chronic inflammatory cardiomyopathy: an expert consensus document. Circ Heart Fail.

[3] Heinsar S, Raman S, Suen JY, Cho HJ, Fraser JF (2021). The use of extracorporeal membrane oxygenation in children with acute fulminant myocarditis. Clin Exp Pediatr.

[4] (2024). Weekly report on COVID-19, influenza and other respiratory viruses (2024) [In Spanish]. https://www.gob.mx/cms/uploads/attachment/file/890801/INFLUENZA_OVR_SE05_2024.pdf.

[5] Butts RJ, Boyle GJ, Deshpande SR (2017). Characteristics of clinically diagnosed pediatric myocarditis in a contemporary multi-center cohort. Pediatr Cardiol.

[6] Rodriguez-Gonzalez M, Sanchez-Codez MI, Lubian-Gutierrez M, Castellano-Martinez A (2019). Clinical presentation and early predictors for poor outcomes in pediatric myocarditis: a retrospective study. World J Clin Cases.

[7] Ghelani SJ, Spaeder MC, Pastor W, Spurney CF, Klugman D (2012). Demographics, trends, and outcomes in pediatric acute myocarditis in the United States, 2006 to 2011. Circ Cardiovasc Qual Outcomes.

[8] Baral N, Adhikari P, Adhikari G, Karki S (2020). Influenza myocarditis: a literature review. Cureus.

[9] Craver RD, Sorrells K, Gohd R (1997). Myocarditis with influenza B infection. Pediatr Infect Dis J.

[10] Tabbutt S, Leonard M, Godinez RI, Sebert M, Cullen J, Spray TL, Friedman D (2004). Severe influenza B myocarditis and myositis. Pediatr Crit Care Med.

[11] Muneuchi J, Kanaya Y, Takimoto T, Hoshina T, Kusuhara K, Hara T (2009). Myocarditis mimicking acute coronary syndrome following influenza B virus infection: a case report. Cases J.

[12] Frank H, Wittekind C, Liebert UG, Siekmeyer M, Siekmeyer W, Schuster V, Kiess W (2010). Lethal influenza B myocarditis in a child and review of the literature for pediatric age groups. Infection.

[13] McCormick AD, Censoplano N, Schumacher KR (2017). Fulminant influenza B myocarditis in a pediatric patient. J Pediatr Intensive Care.

[14] Piccininni JA, Richmond ME, Cheung EW, Lee TM, Law SP, Addonizio LJ, Zuckerman WA (2018). Influenza myocarditis treated with antithymocyte globulin. Pediatrics.

[15] Arguello-Borbón K (2021). Sudden death due to acute myocarditis associated with influenza type B virus. Case report [Article in Spanish]. Med Leg Costa Rica.

[16] Puzelli S, Facchini M, Piacentini S (2024). Characterization of an influenza B virus isolated from a fatal case of myocarditis in a pediatric patient in Italy. J Infect Public Health.

[17] Tian F, Xiao Y, Peng Z (2024). Fulminant myocarditis caused by influenza B virus in a male child: a case report and literature review. J Cardiothorac Surg.

[18] Senekovič Kojc T, Marčun Varda N (2022). Novel biomarkers of heart failure in pediatrics. Children (Basel).

[19] Law YM, Lal AK, Chen S (2021). Diagnosis and management of myocarditis in children: a scientific statement from the American Heart Association. Circulation.

[20] Pei L, Yang N, Yang YH, Guo ZY, Xu W, Liu CF (2015). Clinical features and prognostic factors in children with fulminant myocarditis [Article in Chinese]. Zhongguo Dang Dai Er Ke Za Zhi.

[21] Sun M, Zong Q, Ye LF, Fan Y, Yang L, Lin R (2022). Prognostic factors in children with acute fulminant myocarditis receiving venoarterial extracorporeal membrane oxygenation. World J Pediatr Surg.

[22] Parissis H, Graham V, Lampridis S, Lau M, Hooks G, Mhandu PC (2016). IABP: history-evolution-pathophysiology-indications: what we need to know. J Cardiothorac Surg.

[23] Dimas VV, Morray BH, Kim DW (2017). A multicenter study of the Impella device for mechanical support of the systemic circulation in pediatric and adolescent patients. Catheter Cardiovasc Interv.

[24] Shugh SB, Tume SC, Bansal N (2024). Transcatheter axial pump use in pediatric patients on veno-arterial extracorporeal membrane oxygenation: an ACTION Collaborative Experience. ASAIO J.

[25] Xiong H, Xia B, Zhu J, Li B, Huang W (2017). Clinical outcomes in pediatric patients hospitalized with fulminant myocarditis requiring extracorporeal membrane oxygenation: a meta-analysis. Pediatr Cardiol.

[26] Tonna JE, Boonstra PS, MacLaren G (2024). Extracorporeal Life Support Organization Registry international report 2022: 100,000 survivors. ASAIO J.

[27] Ohki S, Hosokawa K, Tomioka S, Matsuoka M, Fushimi K, Matsuda S, Shime N (2021). Pediatric fulminant myocarditis in Japan: a retrospective nationwide database study of hospital volume, management practices, and mortality. Pediatr Crit Care Med.

